# Smoking prevalence among tuberculosis patients: A crosssectional study in Bangladesh and Pakistan

**DOI:** 10.18332/tid/125452

**Published:** 2020-08-26

**Authors:** Anna-Marie Marshall, Deepa Barua, Alex Mitchell, Ada Keding, Rumana Huque, Amina Khan, Raana Zahid, Omara Dogar, Kamran Siddiqi

**Affiliations:** 1 Department of Health Sciences, University of York, York, United Kingdom; 2 Ark Foundation, Dhaka, Bangladesh; 3 The Initiative, Islamabad, Pakistan

**Keywords:** tobacco, tuberculosis, cessation, survey

## Abstract

**INTRODUCTION:**

Smoking has a negative impact on TB outcomes. We estimated the proportion of TB patients who smoke and are willing to quit in two high TB burden countries, Bangladesh and Pakistan.

**METHODS:**

A cross-sectional survey was conducted among TB patients to assess their eligibility and recruit them to a smoking cessation randomized controlled trial. Adults diagnosed with TB were recruited from 32 health facilities in Bangladesh and Pakistan. Data on smoking behaviour and willingness to quit were collected and analysed.

**RESULTS:**

In total, 13934 TB patients completed the survey between June 2017 and April 2018. The prevalence of smoking in these TB patients was estimated to be 22.5% (95% CI: 21.8, 23.2). Moreover, the prevalence of smoking in TB patient population was 8% (RR=1.49; 95% CI: 7.1–8.9; p<0.01) and 8.3% (RR=1.24; 95% CI: 7.3–9.4; p<0.01) higher than smoking prevalence in the general population in Bangladesh and Pakistan, respectively. Among TB patients who smoke, 97.7% (95% CI: 97.2–98.2) were willing to quit.

**CONCLUSIONS:**

The estimated prevalence of smoking was higher in TB patients than the general population; however, a vast majority of TB patients who smoke were willing to quit.

## INTRODUCTION

Tobacco and tuberculosis (TB) are two of the greatest threats to health worldwide and are both prevalent in many low- and middle-income countries (LMICs). In 2018, TB affected approximately 10 million people worldwide and caused almost 1.5 million deaths^1,2^. The highest number of TB cases were reported in South-East Asia (44%), with Bangladesh (4%) and Pakistan (6%) combined accounting for 10% of the estimated global incidence^3,4^. Both countries are listed in the top 30 high TB burden countries^5^. Bangladesh has a TB incidence of 221 cases per 100000 population. Its national TB control programme offers treatment coverage for 75% of its population and has a treatment success rate of 94%^1^. In Pakistan, TB incidence is 265 cases per 100000 population. TB treatment coverage extends to 64% of its population with a success rate of 93%^1^. It is estimated that TB incidence in Bangladesh and Pakistan will cost the countries 22 and 14 billion US$, respectively, between 2015 and 2030^6^.

Approximately 1.3 billion people smoke tobacco worldwide, 80% of which reside in LMICs such as Bangladesh and Pakistan, where the burden of TB is also high^7^. The prevalence of daily smoking (those who currently smoke tobacco on a daily basis) in the general population according to the most recent Global Adult Tobacco Survey (GATS) reports (GATS 2017 for Bangladesh and 2014 for Pakistan) is 16.4% (33.1% of males and 0.7% of females) and 11.5% (20.6% of males and 2% of females) in Bangladesh and Pakistan, respectively^8,9^.

Smoking has a negative impact on TB outcomes. Smoking is a risk factor for acquiring TB infection^10^ and for the development of TB disease^11^, and is predicted to cause 18 million additional cases of TB by 2050^12^. Moreover, continuing to smoke tobacco after being diagnosed with TB is associated with poorer TB outcomes. For example, smoking exacerbates TB symptoms^13^, is associated with poor treatment outcomes and medication adherence^14,15^, higher risk of TB relapse/recurrence^13,16^, TB drug resistance^17^, increased TB transmission^18^, and TB related deaths^12^. Smoking causes approximately 15–20% of TB related deaths^19^. Due to the detrimental impact that smoking tobacco may have on TB control and targets to end TB, the World Health Organization (WHO) recommends that tobacco smoking is tackled within the framework of TB management and care^20^.

The adverse interaction between TB and tobacco is a global concern and it is therefore important to know the prevalence of smoking in TB patients, particularly in high burden TB countries. The estimates for smoking prevalence in the general population are available for countries with a high TB burden. However, smoking prevalence estimates in the TB patient population are not available for countries such as Bangladesh and Pakistan. This information would allow TB programmes to plan support for TB patients in helping them to quit smoking and would also provide useful information to policy makers and clinicians, within the context of both TB and tobacco smoking prevention. We, therefore, aimed to estimate the prevalence of tobacco smoking amongst TB patients in Bangladesh and Pakistan; we also estimated the proportion of TB patients who are willing to quit smoking.

## METHODS

### Design

A cross-sectional survey was conducted among TB patients to assess their eligibility and recruit them to a smoking cessation randomized controlled trial^21^. The trial assessed the effectiveness and cost-effectiveness of cytisine plus behavioural support compared with placebo plus behavioural support.

### Setting

A purposive sample of health facilities offering TB services in Bangladesh and Pakistan were recruited in accordance with the respective National TB Control Programmes (NTP). Data were collected during the screening process of the above stated trial, for which specific resources were required (feasibility of achieving recruitment targets, TB patient turnover of 200 per month, adequate resources, sites were functioning, designated TB diagnostic centres approved by NTP). The 32 health facilities (17 sub-district hospitals in Bangladesh and 15 secondary care hospitals in Pakistan) were recruited across both urban and rural areas. Recruited sites provided a signed approval letter, a copy of the institutional Review Board approval in line with local regulatory requirements, a completed delegation of responsibilities log, and contact details, before data collected commenced.

### Participants

The survey participants were adults recruited nonrandomly (newly diagnosed TB patients were approached) from the selected health facilities with the help of doctors, DOTS (Directly Observed Treatment, Short Course) providers, community health workers, and laboratory technicians. Eligibility criteria included adult patients with TB, who were diagnosed routinely, within the past 4 weeks in Bangladesh and Pakistan, by methods such as sputum tests, X-rays and GeneXpert, and who were registered with the NTP.

### Measures

Data were gathered using an eligibility assessment form for the above-mentioned trial. The screening form included information on TB and smoking, including TB type (pulmonary or extra pulmonary), and smoking behaviour (if they smoke any form of tobacco, including waterpipe) on a daily basis. Patients were categorised as daily smokers if they had smoked tobacco in 25 out of the past 30 days^22^. Patients were also asked if they are willing to quit. All responses were ‘yes’ or ‘no’.

### Data collection

The survey was administered face-to-face by trained site research assistants at the health facilities, between June 2017 and April 2018. The survey contained no personal information that could be used to identify the patient. Their TB registration number was used as a method of identification. Ethical approval was obtained from the University of York Research Governance Committee and local ethics committees from Bangladesh and Pakistan.

### Statistical analysis

The prevalence of smoking in TB patient population and the 95% confidence intervals (CI) were presented as percentages. Smoking prevalence overall and within subgroups were formally compared to prevalence in the general population via hypothesis testing, taking the relevant GATS point estimate of smoking prevalence as the null value.

The method for GATS data collection is standard across countries and clearly explained by Kalsbeek et al.^23^. In both countries people aged ≥15 years were included in the survey. Participants were recruited using a multi-stage stratified cluster sampling method; this included both males and females, and a sample of rural and urban households. In Pakistan, data were collected in 2014 (which are the most recent available data) sampling 9856 households with 7831 participants completing the survey^9^. In Bangladesh, data were collected in 2017 (again the most recent data), 14880 people were surveyed and 12783 responded^8^.

Smoking status in the screened population was determined by the question: ‘Has the participant been smoking any form of tobacco on a daily basis?’, for which the answers ‘Yes’ or ‘No’ were possible. This was compared to smoking status in the GATS data, which was determined by the question: ‘Do you currently smoke tobacco on a daily basis, less than daily, or not at all?’, for which the answers were ‘daily’, ‘less than daily’, ‘not at all’, ‘don't know’, and ‘refused’. Participants for whom smoking status was not completed were excluded when estimating the prevalence of smoking in the screened population and subgroups.

Subgroup comparisons of smoking prevalence within the screened population, were carried out by country and gender and compared to the relevant GATS estimate. For each subgroup, the difference in prevalence and risk ratio (using the relevant GATS estimate as the comparator) were estimated, along with the corresponding 95% CI and p-value. This analysis was repeated within the subgroup of patients who only had pulmonary TB.

Willingness to quit smoking tobacco was determined by the question: ‘Is the participant willing to quit smoking tobacco?’, for which the answers ‘Yes’ or ‘No’ were recorded. The prevalence of willingness to quit smoking was estimated overall and within each country, along with the corresponding 95% CI and p-value. Participants for whom the willingness to quit status was missing were excluded from the analysis.

## RESULTS

In total, 13934 patients, between June 2017 and April 2018, were screened for inclusion in the trial and therefore took part in the survey. Of those screened, 13906 (99.8%) reported their smoking status and were included in the analyses (Bangladesh 8083 [99.7%]; Pakistan 5823 [99.9%]). Of the 13906 with recorded smoking status, 8447 (60.7%) were male.

### Smoking prevalence

The prevalence of smoking in the screened population was estimated to be 22.5% (95% CI: 21.8–23.2). Table 1 displays the smoking prevalence overall and within subgroups compared to the relevant prevalence obtained from the GATS survey in 2014 for Pakistan and 2017 for Bangladesh. The prevalence of smoking in the screened TB patient population in Bangladesh was 8% (RR=1.49; 95% CI: 7.1–8.9; p<0.01) higher than smoking in the general population. The prevalence of smoking in the screened TB patient population in Pakistan was 8.3% (RR=1.24; 95% CI: 7.3–9.4; p<0.01) higher than smoking in the general population.

**Table 1:** Smoking prevalence in TB patients and their subgroups compared with that in the general population

	*Number of smokers/number in group*	*Prevalence % (95% CI)*	*Absolute difference in prevalence % (95% CI)*	*Absolute difference in prevalence % (95% CI)*	*Relative risk ratio (95% CI)*	*P*
**All screened participants**	3127/13906	22.5 (21.8–23.2)	NA	NA	NA	NA
**Bangladesh participants**	1972/8083	24.4 (23.5–25.3)	16.4	8.0 (7.1–8.9)	1.49 (1.43–1.54)	<0.01
Males	1961/4760	41.2 (39.8–42.6)	33.1	8.1 (6.7–9.5)	1.24 (1.20–1.29)	<0.01
Females	11/3323	0.3 (0.1–0.5)	0.7	-0.4 (-0.6– -0.2)	0.43 (0.14–0.71)	0.01
**Pakistan participants**	1155/5823	19.8 (18.8–20.9)	11.5	8.3 (7.3–9.4)	1.72 (1.63–1.82)	<0.01
Males	1126/3687	30.5(29.1–32.0)	20.6	9.9(8.5–11.4)	1.48 (1.41–1.55)	<0.01
Females	29/2136	1.4 (0.9–1.8)	2.0	-0.6 (-1.1– -0.2)	0.70 (0.45–0.90)	0.03
**All pulmonary TB participants**	2851/11207	25.4 (24.6–26.3)	NA	NA	NA	NA
**Bangladesh pulmonary TB**	1725/6100	28.3 (27.1–29.4)	16.4	11.9 (10.7–13.0)	1.72 (1.65–1.79)	<0.01
Males	1720/3848	44.7 (43.1–46.3)	33.1	11.6 (10.0–13.2)	1.35 (1.30–1.40)	<0.01
Females	5/2252	0.2 (0.0–0.4)	0.7	-0.5 (-0.7– -0.3)	0.29 (0.0–0.57)	<0.01
**Pakistan pulmonary TB**	1126/5107	22.0 (20.9–23.2)	11.5	10.5 (9.4–11.7)	1.91 (1.82–2.02)	<0.01
Males	1098/3291	33.3 (31.8–35.0)	20.6	12.7 (11.2–14.4)	1.62 (1.54–1.70)	<0.01
Females	28/1816	1.5 (1.0–2.1)	2.0	-0.5 (-1.0–0.1)	0.75 (0.50–1.05)	0.16

Of the 13906 participants with a non-missing smoking status, 11207 (80.6%) had pulmonary TB. The prevalence of smoking in participants with pulmonary TB was estimated to be 25.4% (95% CI: 24.6–26.3). Table 1 displays the smoking prevalence overall and within subgroups compared to the relevant prevalence obtained from the GATS survey.

### Willingness to quit

Of the 3127 participants who smoked, 3109 (99.4%) had information on their willingness to quit smoking tobacco and were included in the overall and subgroup willingness to quit prevalence analyses (Bangladesh 1959 [99.3%]; Pakistan 1150 [99.6%]). The prevalence of willingness to quit in the population of all smokers was estimated to be 3037/3109, 97.7% (95% CI: 97.2–98.2). In Bangladesh 1939/1959, 99.0% (95% CI: 98.5–99.4) and in Pakistan 1098/1150, 95.5% (95% CI: 94.3–96.7) of TB patients were willing to quit smoking.

## DISCUSSION

Our study found that the overall prevalence of daily smokers in our sample of TB patients in both countries (24.4% for Bangladesh and 19.8% for Pakistan) was significantly higher (8.0% for Bangladesh and 8.3% for Pakistan) than the prevalence of smoking in the general population (16.4% for Bangladesh and 11.5% for Pakistan). These findings are consistent with studies conducted in other high burden TB countries, such as India, China and South Africa, which have observed a higher prevalence of tobacco smoking in TB patients compared with smoking prevalence in the general population^24-26^. In India, among newly diagnosed TB patients, smoking prevalence was 31.9% in males, which is higher than the general population in South India (25%)^24^. In a study conducted in South Africa, the prevalence of smoking in TB suspects was 57% (63% for males and 44% for females), which was higher than the population estimate of 35% and 10% for males and females, respectively^25^. A further study in China reported a smoking prevalence of 54.6% in TB cases^26^, which was also higher than smoking in the general population, which is approximately 28.1%^27^.

When only pulmonary TB patients were included in the analysis the prevalence rose to 28.3% and 22.0% for Bangladesh and Pakistan, respectively, which is a significant increase of 11.9% for Bangladesh and 10.5% for Pakistan compared to the smoking in the general population. The subgroup analysis by gender also revealed that the prevalence of smoking in male TB patients in both countries was significantly higher than smoking prevalence in the general male population (8.1% and 9.9% higher for Bangladesh and Pakistan, respectively), whilst the prevalence of smoking in female TB patients in the screened population was lower than the prevalence of smoking in the general female population (-0.4% and -0.6% for Bangladesh and Pakistan, respectively).

Smoking is a risk factor for acquiring TB infection^10^, with an RR estimate of 1.73^28^, and for the development of TB disease^11^, with estimates ranging from 2.33 to 2.66^28^. This could explain the greater prevalence of smoking amongst TB patients compared with the prevalence of smokers in the general population. However, there are other potential factors that could explain the high prevalence. Both TB^29,30^, and tobacco use^31^, are more prevalent in those from a low socioeconomic background. Therefore, poor socioeconomic status could be a confounding factor and explain a higher smoking prevalence in TB patients.

Given the detrimental impact that tobacco smoking has on TB outcomes, the high prevalence of tobacco smoking in male TB patients is a worrying finding. These patients may suffer worse TB symptoms^13^, worse treatment outcomes^14,15^, a higher risk of TB relapse/reoccurrence^13,16^, greater risk of TB drug resistance^17^, and a greater risk of TB related death^12^. This also has implications for the wider population, and could increase the spread of TB, as smoking is also associated with increased TB transmission^18^.

## Strengtsechs and limitations

Our large-scale survey included 32 health facilities and 13934 registered TB patients. Many of the TB clinics with high patient load in both counties were surveyed, including both rural and urban areas. As all TB patients are registered in Pakistan and Bangladesh, and this study did include many of the major TB hospitals in both counties, from which we screened all newly diagnosed TB patients, it is likely to be representative of the TB population in these countries. However, it must also be noted that, given that the purpose of screening was for recruitment of smokers for a randomized controlled trial, the sites were chosen on the basis that they had suitable resources to conduct the trial and were not chosen at random. This purposive sampling method may not provide an accurate estimate of smoking prevalence as would population based random sampling, which was employed in the GATS survey. This should be considered when interpreting the findings of this survey.

This study has some further limitations. Our sample was 60% male; therefore, it is likely to give a higher estimate than the general population in which the male population is 50%. Further to this, our data reporting female smokers could be an underestimate. This is due to the socially undesirable nature of tobacco smoking amongst females in areas of South-East Asia^24^. This could result in underreported smoking rates, both in our screened population and in the GATS survey. The smoking rates for females should therefore be interpreted with caution. An additional limitation relates to the data collection methods. Self-report methods were employed, rather than a biological maker such as cotinine or expired CO, therefore, the accuracy associated with using self-reports should be considered when interpreting the findings. Further to this, the screening forms were concise and short to ensure that they could be administered promptly in a healthcare setting. Therefore, data that would have been useful to collect were omitted, such as: the number of cigarettes/bidi/waterpipe smoked, length of time smoking, age, and socioeconomic status (this information is available for trial participants, see Supplementary file Table S1). Future work in this area should collect these data and explore differences between smoking and non-smoking TB patients. Lastly, there are some differences between the methodology in our survey and that of the GATs survey data set, which we used as a comparator. Data collection in our survey was conducted between June 2017 and April 2018, and GATS data for Pakistan and Bangladesh were collected in 2014 and 2017, respectively. The 3-year differences in the time of data collection for Pakistan should be considered when interpreting these findings.

This survey provides useful information for policy makers and clinicians within the context of both TB and tobacco prevention. These findings also further highlight the need to address tobacco smoking in TB patients, which could be carried out by specifically targeting smokers with TB, providing them with smoking cessation support within routine TB care. This supports recommendations to integrate smoking cessation within TB care, previously suggested by Siddiqi and Dogar^32^. Doing so could result in sizable health benefits, not only for TB patients but also for the wider society. This has the potential to make a large impact on reducing the burden of TB, and to help reach targets specified by the WHO to end the TB epidemic^2^.

A very high proportion (97.7%) of TB patients reported that they were willing to quit smoking (99.0% in Bangladesh and 95.5% in Pakistan). We must bear in mind that self-reported willingness to quit may not translate to a quit attempt. We must also be aware that a response bias is always a possibility when collecting self-reported data in a healthcare setting. However, the fact that such a large proportion of TB patients stated that they were willing to quit tobacco smoking is a very positive step towards smoking cessation in this population. From previous research, we know that those who are more motivated to quit are more likely to make a quit attempt^33-36^. A study conducted in India found that 87% of smokers actually quit smoking after TB diagnosis^37^, and a study conducted in China found that TB patients were more receptive to quitting advice during TB treatment^38^. In this case, those who are willing to quit tobacco, because of the impact of tobacco on TB outcomes, may be more likely to quit.

This teachable moment should be utilized. Offering smoking cessation within a population willing to quit smoking has been shown to achieve high quit rates^39^. By integrating smoking cessation within TB care, patients can be supported to quit at this opportune moment. The high willingness to quit, together with evidence for the success of brief smoking cessation behavioural support interventions for TB patients^40^, could have huge benefits for smoking TB patients, improving TB outcomes, and reducing mortality.

## CONCLUSIONS

The estimated prevalence of smoking in male TB patients was higher than among males in the general population; however, most TB patients who smoke tobacco were willing to quit. Taking this into consideration, together with the huge health risk posed by the interaction between TB and smoking, tobacco control efforts and resources should also focus on supporting TB patients to quit.
