# Supplementary material for: Smoking prevalence among tuberculosis patients: A crosssectional study in Bangladesh and Pakistan
Source: Tob Induc Dis. 2020 Aug 26;18:70. doi: 10.18332/tid/125452 (PMC7485439; doi:10.18332/tid/125452)
Supplement: Supplementary file 1 [file TID-18-70-S1.pdf]

Supplementary Table S1: Sample characteristics of those who took part in the subsequent randomised controlled trial.

**Characteristic**	**Overall (N=2472)**
Age (years) [a]	42.5±14.3
Male [b]	2448 (99)
Body Mass Index (BMI) [a c]	18.5±3.2
Marital status [b]	
Single	318 (13)
Separated	8 (0.3)
Married	2111 (85)
Divorced	4 (0.2)
Widowed	31 (1)
TB Score [b d]	
Severity Class I	264 (11)
Severity Class II	1157 (47)
Severity Class III	1022 (41)
Severity Class IV	29 (1)
Forms of tobacco use (combinations possible) [b]	
Cigarettes	2301 (93)
Bidi	259 (10)

Hookah	94 (4)
Electronic cigarettes	3 (0.1)
Smokeless tobacco	170 (7)
Other	63 (3)
Cigarettes smoked per day [a e]	11.1±8.6
Duration of smoking (years) [a]	23.4±14.0
Ever attempted to quit in the past [b]	654 (26)
Strength of urges to smoke in past 24 hours (0- not at all to 5 - all the time) [a f]	2.8±1.1
Tobacco dependence (time to first daily tobacco smoke) [b g h]	
within 5 minutes	680 (28)
within 5 to 30 minutes	844 (34)
within 31 to 60 minutes	437 (18)
after 60+ minutes	508 (21)

a Mean ± SD

b N (%)

c Missing BMI: n=3;

d TB score was based on the presence of TB related signs and symptoms (e.g. chest pain) and being underweight, with a score of 0 to 8, a higher score indicating a greater number of signs and symptoms present

e Missing cigarettes smoked per day: n=175;

f Missing strength of urges to smoke: n=1;

g Tobacco dependence was measured using the second component of the Heaviness to Smoke Index: 'Time to first use of tobacco product after waking'

h Missing tobacco dependence: n=3

© 2020 Marshall A.M. et al.
